# The dynamic reconstruction of the medial patellofemoral ligament shows good subjective outcomes but high rates of recurrent instability: a systematic review and meta-analysis

**DOI:** 10.1530/EOR-2024-0179

**Published:** 2025-10-01

**Authors:** Jonas Eck, Guido Schwarzer, Andreas Frodl, Andreas Fuchs, Tayfun Yilmaz, Hagen Schmal, Kaywan Izadpanah, Markus Siegel

**Affiliations:** ^1^ Department of Orthopedics and Traumatology, Freiburg University Hospital, Freiburg, Germany; ^2^ Institute of Medical Biometry and Statistics, Faculty of Medicine and Medical Center – University of Freiburg, Freiburg, Germany

**Keywords:** patellar instability, medial patellofemoral ligament, MPFL, reconstruction, dynamic reconstruction, knee surgery

## Abstract

**Purpose:**

- The surgical reconstruction of the medial patellofemoral ligament (MPFL) is a commonly used treatment for recurrent patellar dislocations. A surgical method which is frequently used is the dynamic reconstruction of the MPFL (dMPFLr), which involves attaching the released end of a hamstring muscle to the medial patella using a pulley. The aim of this systematic review and meta-analysis is to evaluate the evidence of this method in terms of patient-reported outcomes, the rate of recurrent instabilities, and other complications.

**Methods:**

- MEDLINE® and Web of Science™ were used to identify eligible studies. We used a random-effects meta-analysis to estimate the pooled rates of the Kujala scores, rates of recurrent instabilities, and complication rates.

**Results:**

- A total of 1,087 studies were screened for eligibility. Six studies, comprising a total of 267 treated knees, met the inclusion criteria and were included. All the publications included were retrospective analyses (level of evidence III). The random-effects model showed an overall mean postoperative Kujala score of 86.74 (95% CI: 79.37–94.11; heterogeneity: *I*^2^ = 94%), a recurrent instability rate of 13% (95% CI: 9–18%; heterogeneity: *I*^2^ = 0%), and additional complications mentioned at a rate of 9% (95% CI: 5–16%; heterogeneity: *I*^2^ = 0%).

**Conclusion:**

- Although good results are achieved with regard to the patient-reported outcomes and complication rates, there is a high rate of recurrent instabilities. Despite these results, dMPFLr can be considered an option for the treatment of skeletally immature patients, as it avoids the need for intraoperative fluoroscopy and the risk of epiphyseal joint injury due to femoral fixation.

## Introduction

The medial patellofemoral ligament (MPFL) is the primary passive stabilizer against lateral dislocation (1). The static reconstruction of the MPFL (static MPFLr, sMPFLr) is a viable and widely used treatment for recurrent patellar dislocations. Most commonly, a tendon graft is fixed to the medial side of the patella and the distal femur. Patellar fixation is most commonly achieved with a suture anchor or by channeling the tendon graft through a bone tunnel and femoral fixation with an interference screw (2). This generates mechanical fixed points of the reconstructed passive stabilizer with little to no flexibility. The femoral fixation is crucial, as suboptimal insertion can correlate with poorer clinical outcomes for this procedure (3). The optimal femoral fixation point has been defined by Schöttle and colleagues (4).

It is challenging to perform sMPFLr in children and skeletally immature adolescents because of the close proximity of the distal femoral physis to the femoral insertion (5). Therefore, alternative techniques have been developed to avoid the disturbance of growth following sMPFLr surgery, for example by choosing a femoral fixation distal to the distal femoral physis (6).

An alternative surgical technique which avoids these problems is dynamic MPFL reconstruction (dMPFLr). It was first used in 1904 and has been further developed, as described in detail by Ostermeier and colleagues in 2007 (7, 8). The principle of dMPFLr is to detach either the semitendinosus or gracilis muscle tendon from the tibial pes anserinus, tunnel it through the medial collateral ligament, and fix it to the medial patella. This leads to medial stabilization without any femoral fixation and therefore a more flexible construct.

Another option which avoids growth plate injuries is to tunnel the semitendinosus tendon through the medial collateral ligament (MCL) while leaving the distal attachment in place and cutting the proximal part of the tendon. This technique was described by Deie and colleagues (9, 10). Alm and colleagues used a similar approach, but instead of the MCL, the adductor magnus muscle was used as a pulley (11).

Although various authors have described these techniques, case numbers are small, and clinical evidence is lacking. The purpose of this review is to summarize the aforementioned surgical techniques, perform a meta-analysis of the existing data in terms of patient-reported outcomes, rates of recurrent instabilities, other complications, and place it in the context of surgical strategies for the treatment of recurrent patellar dislocations.

## Methods

This systematic review was conducted and reported in accordance with the Preferred Reporting Items for Systematic Reviews and Meta-Analyses (PRISMA) Statement (12). This study is registered at the International prospective register of systematic reviews ‘PROSPERO’ (ID: CRD42024588806).

Studies were included if the surgical method was described clearly as using one detached end of a hamstring muscle tendon while the other end remained intact and if the population was at least *n* = 4. The detached tendon then had to be fixed to the patellar bone after tunneling it through a soft tissue pulley. In all but one of the studies included, patellar fixation was performed by pulling the free tendon through a drill hole and a subperiosteal suture. Only Alm and colleagues described patellar fixation using a suture anchor (11). For inclusion, studies had to provide clinical follow-up data and be published in English. The studies were divided into two groups, defined by the site of muscle release (distal versus proximal release). One study by Deie and colleagues in 2003 had to be excluded because the patients included were also represented in a study by the authors in 2005 (9, 10).

Studies were searched for in the Web of Science^TM^ and MEDLINE® ALL, using the same search terms but customized for each database (Table 1). The final search was performed on January 12, 2024. Studies were screened by two reviewers (JE and MS) independently. References in the studies included were additionally screened.

**Table 1:** Search strategy by database.

Database	Search strategy
MEDLINE® ALL	((‘mpfl’ or ‘medial patellofemoral ligament’) and (‘instability’ or ‘dislocation’) and (‘reconstruction’ or ‘dynamic’).ab,ti
Web of science ™	AB=((‘mpfl’ OR ‘medial patellofemoral ligament’) AND (‘dislocation’ OR ‘instability’) AND (‘reconstruction’ OR ‘dynamic’)) OR TI=((‘mpfl’ OR ‘medial patellofemoral ligament’) AND (‘dislocation’ OR ‘instability’) AND (‘reconstruction’ OR ‘dynamic’)

Both reviewers collected the available data from each of the studies included. Epidemiological data (e.g. number of patients/knees, age, and sex), objective outcomes (e.g. complications, recurrent dislocation/instability), and patient reported outcome measures (PROMs, e.g. VAS pain, Kujala, IKDC, Lysholm, Tegner) were taken into account. If not described explicitly, data were counted as missing. Due to the different times at which the various parameters were collected in the individual studies, the data from the latest follow-up were analyzed in each case. The Coleman Methodology Score (CMS) was used to assess methodological quality, and the ROBINS-I tool was used to assess the risk of bias of each of the studies included (13, 14).

### Statistical analysis

A statistical analysis was performed using Excel Version 2406 Build 16.0. Meta-analyses were done with the open statistical environment R (R Studio Version 1.2.5033) using the R package meta (15). Study characteristics and patient demographics were described using statistics such as weighted averages and percentages.

We used a random-effects meta-analysis to estimate the pooled mean Kujala scores and rates of recurrent instabilities and other complications for the different types of therapy, and created forest plots to show the results. The between-study variance was estimated by the restricted maximum likelihood (REML) method, and heterogeneity was expressed as I-squared. A *Q* test for heterogeneity was used to test for subgroup differences with a pooled estimate of the between-study variance in subgroups (16).

## Results

**Figure 1:**
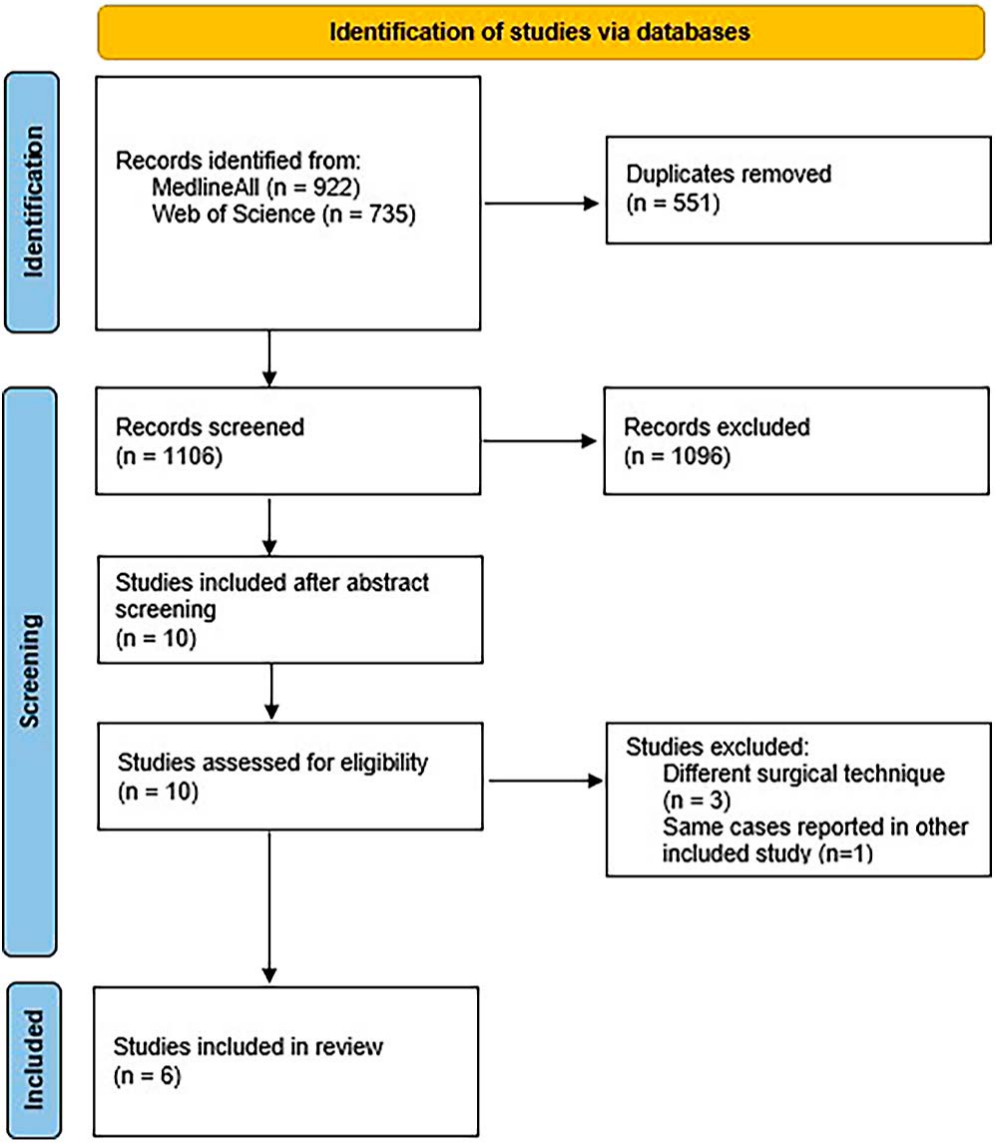
Selection of studies (based on the PRISMA 2020 flow diagram (12)).

Studies were screened for eligibility, as shown in Fig. 1. A total of six studies were included in this meta-analysis, the characteristics of which are shown in Table 2 (8, 9, 11, 17, 18, 19). Coleman scores are rather low, ranging from 33 to 75, with only four of the six studies scoring over 50 points (out of 100). Results of the risk of bias assessment using the ROBINS-I tool are shown in Table 3. Serious risk of bias has to be assumed for each study.

**Table 2:** Characteristics of studies included. Dispersion is presented as either a range or a standard deviation (SD). CMS (Coleman Methodology Score): a point-based scoring system used to systematically assess the methodology of the included studies. The CMS consists of ten categories, and the overall score ranges from 0 (worst) to 100 (best).

Study	Year of publication	Number of patients/knees		Mean age [years]	Mean follow-up	CMS
		All (knees)	F/M (knees)			
Distal release						
Ostermeier *et al.* (8)	2007	14 (14)	-	-	13 (8–27) months	33
Becher *et al.* (18)	2014	15 (15)	10/5 (10/5)	22.1 ± 3.7	26 ± 0.6 months	61
Kiran *et al.* (17)	2015	4 (4)	1/3 (1/3)	25 (18–29)	24 (24–24) months	41
Horstmann *et al.* (19)	2021	152 (158)	−/− (106/52)	22.5 ± 8.1	5.4 ± 1.8 years	59
Proximal release						
Deie *et al.* (9)	2005	43 (46)	34/9 (−/−)	19.2 (6–43)	9.5 (5–12) years	58
Alm *et al.* (11)	2017	28 (30)	17/11 (−/−)	15.1 (11–17)	25.6 (12–43) months	75

F/M, females/males.

**Table 3:** Risk of bias (ROB) assessment using the ROBINS-I tool (14).

ROB assessment criteria	Ostermeier *et al.* (8)	Becher *et al.* (18)	Kiran *et al.* (17)	Horstmann *et al.* (19)	Deie *et al.* (9)	Alm *et al.* (11)
Confounding	Serious	Serious	Serious	Serious	Serious	Serious
Selection of participants	Moderate	Moderate	Moderate	Moderate	Moderate	Moderate
Classification of interventions	Low	Low	Low	Low	Low	Low
Deviations from intended intervention	Low	Low	Low	Low	Low	Low
Missing data	Moderate	Low	Low	Low	Low	Low
Measurement of outcomes	Serious	Serious	Serious	Serious	Serious	Serious
Selection of the reported result	Low	Low	Low	Low	Low	Low
Overall ROB	Serious	Serious	Serious	Serious	Serious	Serious

**Figure 2:**
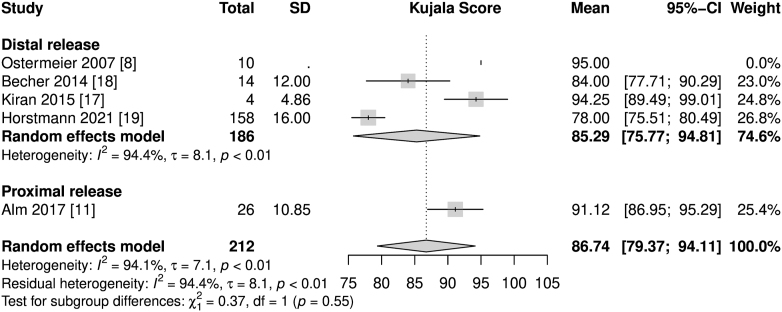
Forest plot of Kujala scores.

All studies used the Kujala score (0–100) as a subjective outcome measure (20). Only Deie and colleagues did not publish exact values (9). Therefore, this study was excluded from the meta-analysis of Kujala scores. Every other study included presented mean Kujala scores postoperatively (Fig. 2). The mean Kujala scores ranged from 78.00 (Horstmann and colleagues) to 95.00 (Ostermeier and colleagues), while showing considerable heterogeneity, *I*^2^ = 94%. The heterogeneity of the Kujala scores could mainly be caused by the variety of mean follow-ups, which ranged from 13 months to 7.4 years.

The Kujala score was the only homogeneously used PROM among the studies included. Mean postoperative pain on a visual analog scale (VAS, 0–10) was reported by Becher and colleagues (1.8), Horstmann and colleagues (1.9), and Alm and colleagues (0.57), resulting in an overall mean postoperative pain of 1.38 (95% CI: 0.48–2.28) under the random-effects model (11, 18, 19).

**Figure 3:**
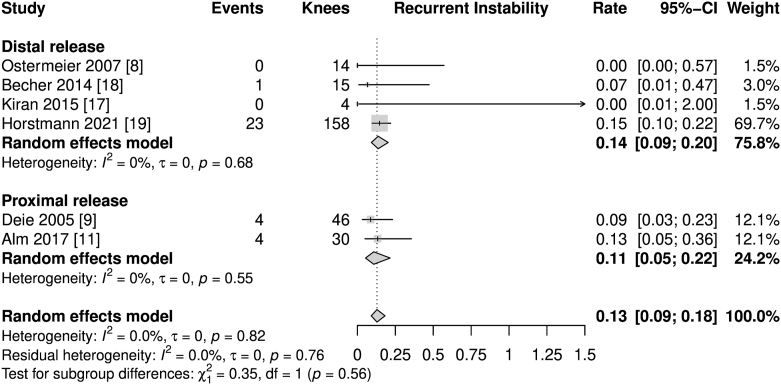
Forest plot of recurrent instabilities.

Recurrent instabilities, including re-dislocations, were reported in each of the studies included as a measure of treatment failure (Fig. 3). The random-effects model showed an overall rate of 13%, with no heterogeneity, *I*^2^ = 0%.

**Figure 4:**
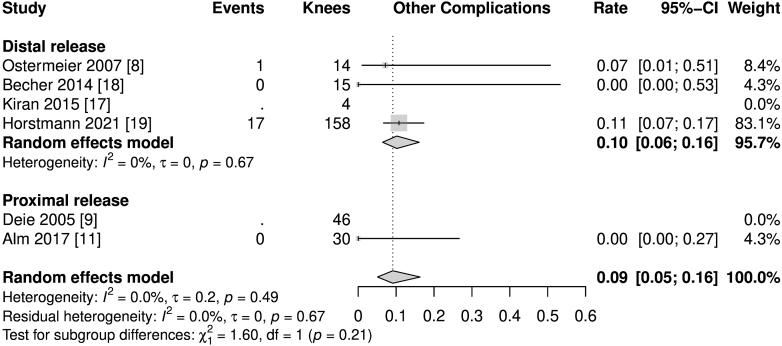
Forest plot of other complications. Comparability of data could be limited due to inconsistent reporting of complications across the included studies.

Whether complications other than recurrent instability occurred was mentioned in four of the six studies (Fig. 4). Alm and colleagues described none, while Ostermeier and colleagues reported one case of a relevant postoperative hematoma (8, 11). Horstman and colleagues delivered a detailed list of complications both related and unrelated to the procedure. Some of these complications were stiffness, hematomas, and infections (19). The overall rate of complications, not including recurrent instabilities, was 9% (heterogeneity: *I*^2^ = 0%).

A test for subgroup differences showed no statistically significant difference regarding any of the outcome parameters analyzed.

## Discussion

The main findings of this study are that dMPFLr leads to good subjective outcomes but is associated with high rates of recurrent instabilities.

With regard to the existing data and literature, dMPFLr seems to be a valid option for the surgical treatment of recurrent patella dislocations, especially for immature patient cohorts. One of the potential advantages of dynamic over static MPFLr is the reduced risk of graft over-tensioning, which causes increased medial patellofemoral contact pressure as well as malpositioning of the femoral insertion (18, 21). Neither plays a role in dMPFLr. Another benefit is the feasibility of radiation-free implantation, eliminating the need for fluoroscopic verification of the femoral drill holes. Furthermore, the risk of injury to the femoral growth plate due to misplaced femoral insertion holes is virtually non-existent, highlighting the value of dMPFLr and techniques without femoral insertion, such as dMPFLr in general, for adolescent patient populations. An alternative surgical technique, of MPFLr without femoral drilling is the Adductor sling technique in which a free tendon graft is looped around the distal tendon of the Musculus adductor magnus and fixated to the medial patella (22). Thus far, few data exist on the clinical outcomes associated with this technique.

The data presented state good postoperative Kujala scores (86.74 pts in the random-effects meta-analysis), which are comparable to the Kujala scores of patients after the commonly performed sMPFLr using autologous hamstring tendon grafts. This is currently the gold standard, with values of 85.79–92.02 pts observed in various meta-analyses (23, 24, 25, 26, 27). Although there are comparable outcomes within subjective parameters, the rate of recurrent patellofemoral instability of 13% is high. It is significantly higher than previously reported rates of recurrent patellofemoral instabilities of 1.2% after sMPFLr (26). This is probably due to the more flexible fixation and must be considered a serious downside of the techniques mentioned without femoral insertion and must be taken into account when planning a patella-stabilizing surgical procedure. However, the overall rate of complications following dMPFLr does not seem to exceed those after sMPFLr with regard to the existing literature (13% recurrent instabilities and 9% other complications vs 26.1% overall cumulative complications) (28).

To date, there have been no studies that have investigated dMPFLr with concomitant procedures, such as tuberosity osteotomy, leg axis osteotomies, or trochleoplasty. The surgical procedure decision should therefore be made after careful consideration of the advantages and disadvantages of the respective procedure and should be individualized. dMPFLr can lead to satisfactory clinical results without intraoperative radiation application and without the risk of epiphyseal injury, and may thus be an option for skeletally immature patients, but no data are yet available on the factors that influence the success rate of this surgical procedure. There is also a lack of studies with the large numbers of cases required to generate good evidence regarding this approach.

Bartsch and colleagues presented a randomized clinical trial protocol in 2022 that proposed a prospective comparison of dynamic and static MPFLr (29). This could deliver an important contribution to the evaluation of dMPFLr.

### Limitations

Some limitations of this meta-analysis include the absence of randomized controlled trials (RCTs) and studies with large patient cohorts. Small patient numbers across studies are major limitations, along with methodological shortcomings, low CMS scores, and high risks of bias. Variability in reported outcome parameters across publications complicates direct comparisons. We conducted tests for subgroup differences to evaluate differential effects for distal and proximal release. For the outcomes recurrent instability and other complications, the statistical power to detect subgroup differences is very low due to small sample sizes and the very low number of events in most studies, resulting in very wide study-specific confidence intervals. Accordingly, no definite statement on subgroup differences is possible for these analyses. Significant disparities in patient numbers in the studies introduce potential bias into the results. Specifically, Horstmann and colleagues’ patient population, comprising the majority in the distal release group, exhibited the lowest mean Kujala scores (78.00) and the highest rate of recurrent instabilities (15%) (19). Ostermeier and colleagues lost four of the 14 patients treated to follow-up with regard to Kujala scores, while Becher and colleagues excluded the Kujala score of a patient who experienced a re-dislocation from the overall mean, potentially inflating scores in small sample sizes (8, 18). Overall, the available data do not definitively establish the long-term outcomes of dMPFLr. Further investigations, particularly prospective RCTs, are essential for a more comprehensive evaluation.

## Conclusion

Although comparable results to the commonly performed sMPFLr using hamstring grafts are achieved with regard to patient-reported outcomes, there is a significantly higher rate of recurrent instabilities. This sincere downside has to be taken into account when considering dMPFLr. Despite these results, dMPFLr can be considered an option for the treatment of skeletally immature patients, as it avoids the need for intraoperative fluoroscopy and the risk of growth plate injury due to femoral fixation.

## ICMJE Statement of Interest

The authors declare that there is no conflict of interest that could be perceived as prejudicing the impartiality of the work reported.

## Funding Statement

This work did not receive any specific grant from any funding agency in the public, commercial, or not-for-profit sector.
